# Optimization of Diclofenac-Loaded Bicomponent Nanofibers: Effect of Gelatin on In Vitro and In Vivo Response

**DOI:** 10.3390/pharmaceutics16070925

**Published:** 2024-07-11

**Authors:** Iriczalli Cruz-Maya, Valentina Cirillo, Janeth Serrano-Bello, Carla Serri, Marco Antonio Alvarez-Perez, Vincenzo Guarino

**Affiliations:** 1 Institute of Polymers, Composite and Biomaterials, National Research Council of Italy, Mostra d’Oltremare, V.le J.F.Kennedy 54, 80125 Naples, Italy; cdiriczalli@gmail.com (I.C.-M.); valentina.cirillo3@gmail.com (V.C.); 2 Tissue Bioengineering Laboratory, Department of Posgraduate Studies and Research (DEPeI), School of Dentistry, Universidad Nacional Autonoma de Mexico (UNAM), Circuito Exterior s/n, Mexico City 04510, Mexico; janserbello@fo.odonto.unam.mx; 3 Department of Medicine, Surgery and Pharmacy, University of Sassari, Via Muroni 23/a, 07100 Sassari, Italy; cserri@uniss.it

**Keywords:** electrospinning, nanofibers, anti-inflammatory drugs, drug delivery, animal model

## Abstract

The use of electrospun fibers as anti-inflammatory drug carriers is currently one of the most interesting approaches for the design of drug delivery systems. In recent years, biodegradable polymers blended with naturally derived ones have been extensively studied to fabricate bioinspired platforms capable of driving biological responses by releasing selected molecular/pharmaceutical signals. Here, sodium diclofenac (DicNa)-loaded electrospun fibers, consisting of polycaprolactone (PCL) or gelatin-functionalized PCL, were studied to evaluate fibroblasts’ in vitro and in vivo response. In vitro studies demonstrated that cell adhesion of L929 cells (≈70%) was not affected by the presence of DicNa after 4 h. Moreover, the initial burst release of the drug from PD and PGD fibers, e.g., 80 and 48%, respectively, after 5 h—combined with its sustained release—did not produce any cytotoxic effect and did not negatively influence the biological activity of the cells. In particular, it was demonstrated that the addition of gelatin concurred to slow down the release mechanism, thus limiting the antiproliferative effect of DicNa, as confirmed by the significant increase in cell viability and collagen deposition after 7 days, with respect to PCL alone. In vivo studies in a rat subcutaneous model also confirmed the ability of DicNa-loaded fibers to moderate the inflammatory/foreign body response independently through the presence of gelatin that played a significant role in supporting the formation of small-caliber vessels after 10 days of implantation. All of these results suggest using bicomponent fibers loaded with DicNa as a valid therapeutic tool capable of supporting the wound healing process and limiting in vivo inflammation and rejection phenomena.

## 1. Introduction

Inflammation is the natural response of tissues to protect organisms from infection and injuries. However, recent studies demonstrated that uncontrolled inflammatory events can negatively influence the mechanisms of tissue repair and regeneration by promoting the rise of chronic diseases (i.e., osteoarthritis, diabetes) [1,2]. Clinical therapies based on the local administration of anti-inflammation drugs (i.e., nonsteroidal) have been efficacious in fighting inflammation. However, the presence of dose-depending side effects (i.e., gastrointestinal problems) was recorded in several cases, thus drastically decreasing the life quality of patients.

During the last few years, a large variety of synthetic or natural polymers have been used to fabricate drug delivery systems to regulate the inflammatory response during tissue regeneration. In the current market, higher diffusion of formulations mainly involves the use of synthetic polymers (i.e., α-hydroxy acids [3], polyanhydrides [4], poly(amides) and (ester amides) [5], poly(phosphor esters) [6], poly(alkyl cyanoacrylates)), basically preferred for their low immunogenicity [7], and controllable hydrolytic degradation profiles that allow us to achieve the desirable pharmacokinetics [8]. In this context, natural polymers, including proteins or polysaccharides, may also be a complementary or alternative solution to synthetic polymers to produce formulations with better drug release tuning and enhanced drug stability.

For this purpose, electrofluid dynamic technologies, including electrospinning, are considered valid techniques to fabricate micro/nanostructured platforms with fully interconnected porosity and a large surface area, suitable for efficiently releasing active molecules at the site of action through balanced diffusion/transport into/through the fiber network [9,10], therefore minimizing the disadvantages of systemic administration [11,12]. Moreover, the fibrillar structure of electrospun fibers facilitates cell adhesion and proliferation, increasing the advantages of their use in biomedical applications [13,14]. Among the broad types of polymers used for the fabrication of drug-loaded electrospun fibers, polycaprolactone (PCL) and gelatin exhibit good biocompatibility for applications in tissue engineering and regenerative medicine [15,16,17], despite some drawbacks, including hydrophobicity, a lack of binding motifs related to PCL [18,19], poor mechanical properties, and in vitro fast dissolution related to gelatin [20,21,22]. In order to overcome these intrinsic limitations of single materials, several studies have proposed to blend PCL and gelatin to merge their main advantages in terms of biomechanical, physical, and biological properties [23,24,25]. However, there is still a significant lack of research specifically aiming to explore the effects of released drugs on in vitro and, especially, in vivo responses. 

From this perspective, sodium diclofenac (DicNa)-loaded electrospun fibers, composed of PCL or PCL functionalized with gelatin, were proposed to investigate the influence of DicNa release—determined by the peculiar physicochemical properties of the fibers—on the in vitro and in vivo response to validate the use of bicomponent fibers as therapeutic systems able to support tissue regeneration and also to resolve the local inflammation phenomena occurring during the regeneration process.

## 2. Materials and Methods

### 2.1. Materials

Poly ε-caprolactone (PCL, Mn 45 kDa), gelatin type B (~225 Bloom from bovine skin in powder form), diclofenac sodium salt (DicNa), methanol and Trifluoroacetic acid (TFA), 2-(N-morpholino) ethanesulfonic acid (MES), EDC ((1-ethyl-3-(3-dimethylaminopropyl)carbodiimide hydrochloride) and N-hydroxysulfosuccinimide (N-NHS), Na_2_HPO_4_, NaCl, and KCl were all purchased from Sigma Aldrich (Milan, Italy). Other compounds, such as 1,1,1,3,3,3-hexafluoro-2-propanol (HFIP) and chloroform (CHCl_3_), were supplied by J.T. Baker (Rodano, Italy). All of the products were used as received without further purification.

### 2.2. Fiber Fabrication

PCL and PCL-Gel nanofibers were fabricated using the electrospinning technique starting from different polymeric solutions: PCL (0.1 g/mL) in 1,1,1,3,3,3 hexafluoroisopropanol (HFIP), or PCL and gelatin at a 50:50 ratio (total concentration 0.1 g/mL) in HFIP. Solutions were mixed with a magnetic stirrer at room temperature and in dry environmental conditions—the humidity degree was less than 50% for about 24 h. In the case of drug-loaded fibers, sodium diclofenac (5 mg/mL) was added about two hours before the complete dissolution of polymers. Each polymer solution was placed in a 5 mL plastic syringe and forced to move through an 18-gauge needle connected to a high-voltage power supply. Fibers were collected onto a ground plate covered with aluminum foil using a commercially available electrospinning setup (Nanon 01, MECC, Fukuoka, Japan), working at 23–26 °C and 40–50% relative humidity degree. In order to better control solvent evaporation, the translation movement of the spinneret was also regulated by setting the translational rate and the length of deposition area. A summary of the process parameters used for the preparation of PCL and PCL/gelatin samples—named as PD and PGD, respectively—is reported in Table 1.

### 2.3. Morphological Studies

Scanning electron microscopy (FESEM, QUANTA200, FEI, Eindhoven, The Netherlands) was used to qualitatively investigate fiber morphology. Images were taken by working at a low voltage—lower than 10 kV—in order to minimize sample burning under the electron beam. All of the samples were dried in the fume hood for 24 h, mounted on metal stubs, sputter-coated with gold palladium, and analyzed under high-vacuum conditions using the secondary electron detector. Then, fiber diameter was measured from selected micrographs using image analysis software (Image J, version 1.39). Fiber mean diameters were calculated from at least 30 measurements from three independent samples. Meanwhile, energy dispersive spectroscopy (EDS) analysis was performed to confirm the encapsulation of the drug by detecting selected chemical elements (Na, Cl). In this case, the samples were not sputter-coated to avoid the comparison of irrelevant peaks in the spectra. 

### 2.4. Thermal Analysis

Thermogravimetric analysis (TGA Q500, TA Instrument, Milan, Italy) was carried out under nitrogen flow within a temperature range from 25 to 600 °C and at a scanning rate of 10 °C/min. Weight loss and derivative functions were grafted versus temperature to analyze changes in the peak shape ascribable to the presence of the drug.

### 2.5. Encapsulation Efficiency

Encapsulation efficiency and drug release profiles were evaluated for different DicNa-loaded nanofibers. In the case of bicomponent fibers, gelatin was preliminarily cross-linked in MES buffer by EDC-NHS with a molar ratio equal to 2, which agrees with previous studies [26]. Briefly, samples with a known mass were dissolved in 1 mL of TFA, and the solution was added dropwise to 20 mL of methanol [27,28], in which the polymer was precipitated and DicNa was dissolved for 4 h. After centrifugation at 4000 rpm for 10 min at 4 °C (Eppendorf Centrifuge 5702 R, Hamburg, Germany) of the methanol solution, the liquid supernatant was collected, and DicNa was quantified by spectrophotometric assay (UV-1800; Shimadzu Laboratory World, Tokyo, Japan) at λ = 323 nm [29,30,31]. A calibration curve calculated the amount of DicNa in a concentration range of 0.5–50 mg/mL (y = 0.0184x + 0.0251 R^2^ > 0.998). The encapsulation efficiency EE% was calculated by Equation (1):

(1) $$EE\%=100\cdot \frac{C_{e}}{C_{T}}$$

where C_e_ is the amount of entrapped drug in the nanofibers and C_T_ is the total amount of the drug used to prepare the nanofibers. The results were recorded as average ± standard deviation for at least three independent batches.

### 2.6. In Vitro Release

The release of DicNa from PD and PGD nanofibers was determined by preliminarily soaking the samples in 10 mL of PBS pH 7.4 buffer solution, and then moving them into a thermostatic incubator for shaking (100 rpm) at 37 °C. At scheduled time intervals, the release medium was withdrawn and replaced with the same volume of fresh medium. The amount of DicNa in PBS was estimated in a concentration range of 0.2–50 mg/mL. For this purpose, a calibration curve was constructed from a series of DicNa solutions with standard concentrations and determined at λ 280 nm (y = 0.0357x + 0.0175 R^2^ = 0.9969) [32,33,34]. Regarding the UV absorbance of PCL and gelatin, previous studies have reported UV-Vis spectra indicating absorbance peaks at approximately 450 nm for PCL and 220 nm for gelatin [35,36].

### 2.7. In Vitro Studies

#### 2.7.1. Cell Culture

In vitro assays were performed using an L929 cell line (fibroblasts derived from mice) as a model according to international standards (ISO-10993-5:2009) [37]. Cells were cultured in a 75 cm^2^ cell culture flask in Dulbecco’s Modified Eagle Medium (DMEM, Sigma-Aldrich, Milan, Italy) supplemented with 10% fetal bovine serum (FBS, Sigma-Aldrich, Milan, Italy), an antibiotic solution (streptomycin 100 μg/mL and penicillin 100 U/mL, Sigma-Aldrich, Milan, Italy), and 2 mM of L-glutamine. The cells were incubated at 37 °C in a humidified atmosphere with 5% of CO_2_ and 95% air. 

For all experiments, PD, PGD, and CTR (no drug) samples were cut and placed into a 96-well cell culture plate and sterilized with a 70% ethanol solution for 30 min; the samples were washed and air-dried. The experiments were conducted in triplicate (n = 3).

#### 2.7.2. Cell Adhesion

For cell attachment, L929 cells were seeded onto PD and PGD fibers at a cell density of 1 × 10^4^ cells/well and allowed to adhere in standard conditions for 4 and 24 h. After incubation, the samples were washed three times with phosphate buffer solution (PBS, Sigma-Aldrich, Milan, Italy) to remove the non-adherent cells. The attached cells were fixed with 4% paraformaldehyde (PFA) and incubated with 0.1% toluidine blue for 3 h. After that time, the excess of dye was removed, and the samples were washed with distilled water. To evaluate cell adhesion, the dye of adherent cells was extracted with 1% sodium dodecyl sulfate (SDS), and the optical absorption was quantified by spectrophotometry at 600 nm with a plate reader (Wallac Victor 1420, PerkinElmer, Boston, MA, USA). The cell culture plate was used as a control. The results are presented as mean ± standard error percentage with respect to the control.

#### 2.7.3. Cell Viability

The cell viability of L929 cells was evaluated by using the Cell Counting Kit-8 (CCK-8, Dojindo Laboratories, Kumamoto, Japan). This assay is based on the reduction of water-soluble tetrazolium salt by the dehydrogenase activity of living cells to give a yellow-color formazan dye, which is soluble in the tissue culture media. L929 cells were seeded at 5 × 10^3^ cells/well on PD and PGD fibers to evaluate viability at 2, 5, 7, 14, and 21 days of culture. After incubation, the samples were incubated with 100 μL of fresh medium containing 10 μL of CCK-8 reagent and incubated for 4 h in standard conditions. After that time, the supernatant was recovered, and absorbance was measured at 450 nm with a plate reader (Wallac Victor 1420, PerkinElmer, Boston, MA, USA). The amount of the formazan dye generated by the dehydrogenases in cells is directly proportional to the number of living cells. The cell culture medium was changed every two days with fresh medium during the experiment. 

#### 2.7.4. In Vitro Collagen Deposition

Sirius red assay was used to evaluate the collagen deposition of cells seeded onto PD and PGD fibers. The samples were placed into a 96-well cell culture plate, and cells were seeded at 1 × 10^4^ per well to perform the assay at 7, 14, and 21 days. L929 cells seeded onto a cell culture plate were cultured in the same conditions and used as the control of the experiment. Cells were fixed with 4% PFA and washed for the Sirius red assay. To quantify the collagen deposition in wells and electrospun fibers, 0.1% of Sirius red dye in a saturated aqueous solution of picric acid was added to samples and incubated for 1 h. After incubation, the samples were washed with 0.01 N of HCl to remove the excess dye. The extraction of stained collagen was obtained with 0.1 N of NaOH. The supernatant was recovered to measure the absorbance at 540 nm. The collagen deposition from L929 cells was normalized with respect to the absorbance values of cells seeded onto the culture plate.

### 2.8. Statistical Analyses

Biological studies were conducted using statistical analyses via one-way analysis of variance, followed by Tukey’s post-hoc analysis. Data are presented as mean ± standard error. *p*-values < 0.05 were considered statistically significant.

### 2.9. In Vivo Studies

#### 2.9.1. Animal Model

An in vivo model was used to analyze the contribution of DicNa-loaded nanofibers to the healing process and control of local inflammation. Before the materials were implanted, they were sterilized using ethylene oxide at the hospital level. In this model, the electrospun material was implanted in the rat’s dorsum as described in the Surgical Procedure Section. 

For the in vivo model, we used 12 male Wistar rats, 18 weeks old, weighing 250 g, and housed at a constant temperature of 24 °C, with a 12 h light to 12 h dark rotation. They were fed standard pellets for rodents (rodent diet 5001), and water was administered ad libitum. The surgical procedure was performed according to what was established by the Internal Committee for the Care and Use of Laboratory Animals of the Dentistry School, with approval number FO-M001-0009-202 and the Mexican legislation NOM-062-ZOO1999. The rats were randomly assigned to three groups of four rats, depending on different periods of time (4, 10, and 21 days).

#### 2.9.2. Surgical Procedure 

The surgical procedures were performed as reported elsewhere [38]. The rats were sedated and tranquilized intramuscularly with Ketamine (80 mg/kg) and Xylazine (10 mg/kg). Subsequently, the back was shaved and the surgical area was prepared by applying an electrolyzed superoxidation solution with a neutral pH containing active chlorine and oxygen at 0.002% (Estericide, Esteripharma, México City, México).

The animals were carefully positioned on a heated operating table, and a precise 2 cm long incision was made. The skin was separated from the subcutaneous tissue using delicate fine tweezers to create a subcutaneous pocket. The sample was inserted as far away from the incision as possible and sutured with 5-0 nylon in the subcutaneous tissue to ensure minimal sample movement. This procedure was repeated for all conditions, resulting in the following: CTR in the upper right quadrant, PD in the lower left quadrant, and PGD in the lower right quadrant, as shown in Supplementary Figure S1.

The animals were closely and attentively monitored throughout the study for general condition, wound appearance, signs of suppuration, pain, or weight loss.

At the end of the experimental periods (4, 10, and 21 days), the animals were euthanized in a CO_2_ chamber with a 70% filling rate. Subsequently, the samples were removed via excisional biopsy, leaving a 2 mm security margin, and placed in 10% formalin for 24 h for fixation. Afterward, serial sections of 5 µm thickness were made and stained with hematoxylin and eosin for evaluation under light microscopy.

## 3. Results and Discussion

**Figure 1:**
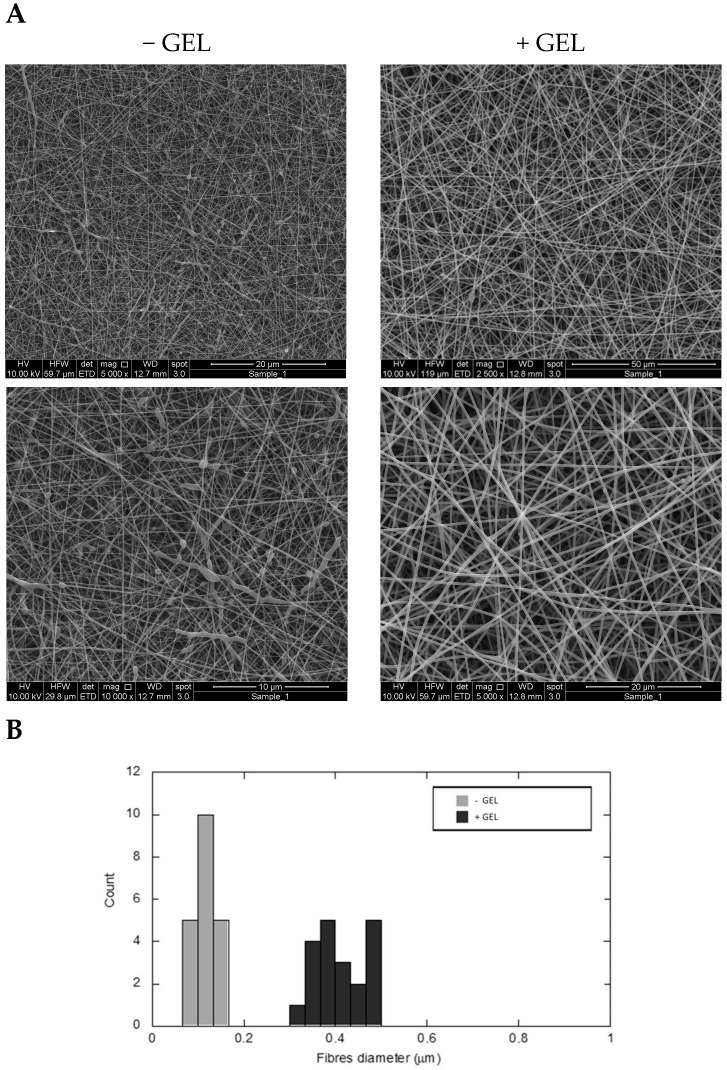
DicNa-loaded electrospun fibers: SEM images of PCL and PCL/gelatin nanofibers at different magnification (**A**) and fiber diameter distribution via image analysis (**B**).

In this study, DicNa-loaded fibrous membranes were fabricated using the electrospinning technique to investigate the therapeutic effect of DicNa on in vitro and in vivo responses. Using PCL and gelatin blending, two different fibers were proposed as drug carriers, PCL nanofibers and bicomponent nanofibers. A preliminary study was performed on setting the process parameters to optimize fiber morphology (Figures S2 and S3). The optimal parameters and process conditions are reported in Table 1. Qualitative and quantitative information on the morphology of selected DicNa-loaded fibers was further investigated (Figure 1). 

The SEM images show a homogeneous spatial distribution of fibers falling in the sub-micron range, randomly distributed in the membrane (Figure 1A). Some beads can be recognized along the PCL fibers (−GEL), probably due to the effect of local inhomogeneity of the drug in the fiber. Likewise, no defects can be distinguished in the case of bicomponent fibers (+GEL), where gelatin plays an active role in fiber stabilization during the electrospinning process, minimizing the varicose effect and fluid dynamic instability phenomena, as reported in previous studies [39]. The distribution of fiber diameters was assessed by using image analysis (Figure 1B). Bicomponent fibers show a broader diameter distribution than PCL ones, with statistical modes of 0.44 μm and 0.18 µm and skewness of 0.16 and 0.05, respectively. 

**Figure 2:**
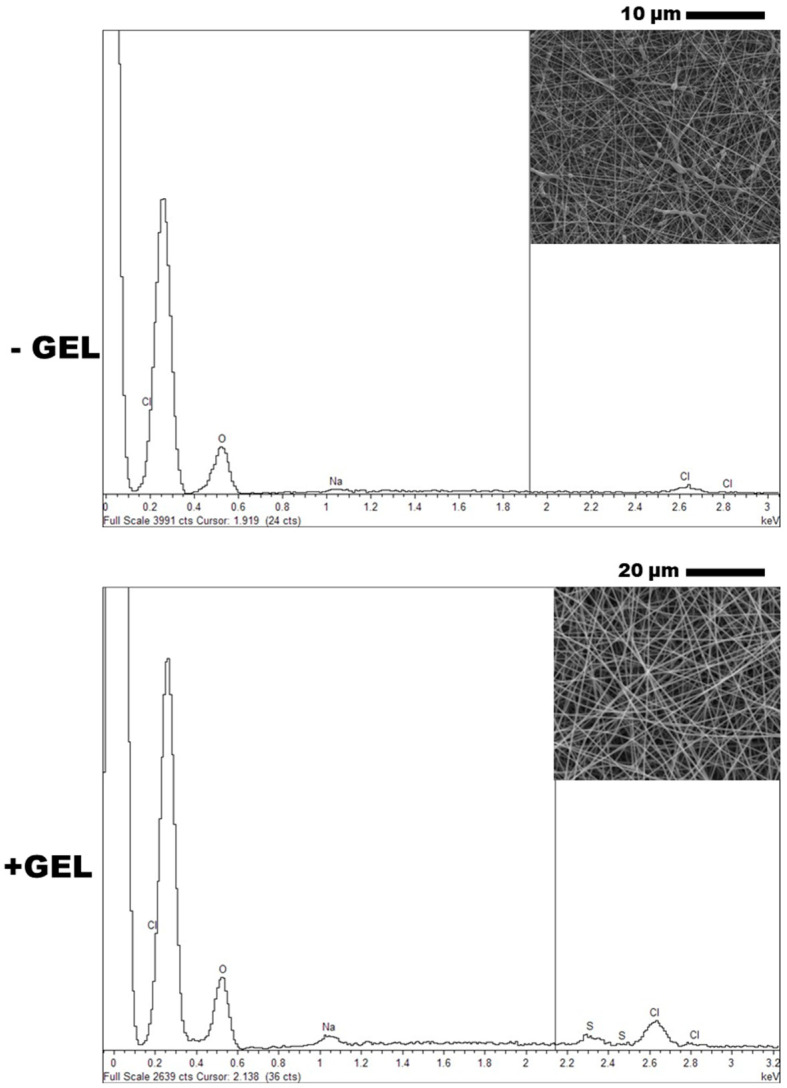
DicNA-loaded electrospun fibers: detection of Na elements via EDS analysis. Detail of fiber morphology shown in square.

The presence of DicNa was preliminary detected by an EDS probe (Figure 2). In both spectra, it was possible to clearly distinguish the peak of the Na element present in the salt, even though it appears to be more evident in the case of bicomponent fibers. Hence, thermogravimetric analyses were carried out to quantify the amount of DicNa in the fibers.

**Figure 3:**
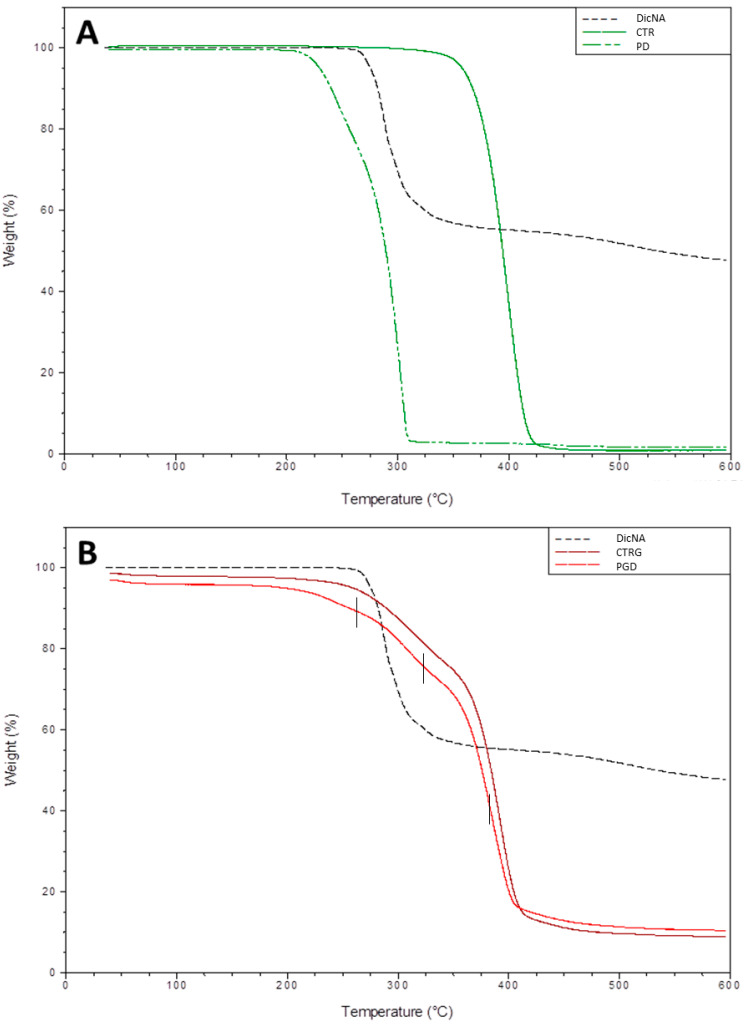
DicNa quantification via TGA analyses of DicNa-loaded (**A**) PCL (PD), and (**B**) PCL/gelatin nanofibers (PGD). DicNa is referred to as the thermogram of the drug, while CTR and CTRG, respectively, are reported as controls for unloaded PCL and PCL/gelatin nanofibers.

Figure 3 shows the thermogram of DicNa-loaded fibers compared to DicNa and unloaded fibers, used as negative controls. In Figure 3A, the PD thermogram highlights a weight decay between 280 °C and 300 °C, related to drug transition, according to the DicNa curve. Through comparison with the CTR curve related to unloaded fibers, it was possible to observe an evident temperature reduction, shown in the curve’s transition—from about 400 °C to 300 °C—ascribable to drug–polymer interactions, in agreement with previous experimental studies reported by Šišková et al. [40]. Noteworthy, this effect was not observed in the case of bicomponent fibers (Figure 3B) due to the contribution of gelatin that tends to entrap the drug through polar interactions. Instead, the PGD curve shows decomposition in three steps ascribed to DicNa, gelatin, and PCL’s weight decays—onset decomposition temperatures of 274 °C, 316 °C, and 386 °C (see Figure 3B), respectively. The change in curve slope—around 360 °C—can be associated with the transition switch from gelatin to PCL, in agreement with data reported in the derivate curves (Figure S4). 

DicNa’s encapsulation efficiency of PD and PGD nanofibers was calculated using spectrophotometric analyses. The EE% was around 99.2 ± 0.2% and 98.8 ± 0.4% in the case of PD and PGD nanofibers, respectively. No significant difference was observed concerning theoretical loading, which agrees with previous studies on equivalent fibers reported in the literature [41].

**Figure 4:**
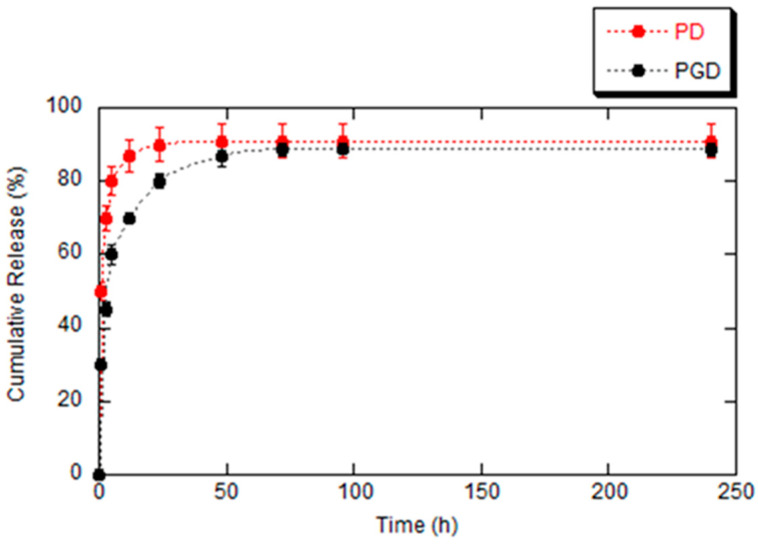
Comparison of cumulative release profiles of DicNa from electrospun fibers in PBS solution. Six independent experiments were performed, and results are expressed as mean values obtained (mean ± SD).

The in vitro release of sodium diclofenac (DicNa) from PD and PGD nanofibers was evaluated at 37 °C in PBS, as illustrated in Figure 4. The release profiles were reproducible, demonstrating the ability of both PD and PGD nanofibers to control and sustain DicNa release. An initial burst release was observed for both nanofibers, with approximately 80% of DicNa released from PD and 48% from PGD. The complete release occurred within approximately 250 h, with notable differences between the two formulations. Specifically, the PD profile exhibited a significant initial burst release of nearly 80% within the first 5 h, followed by a constant and continuous release of DicNa. In contrast, the PGD nanofibers demonstrated a less pronounced burst release, about 25% lower than that of the PD nanofibers at 5 h, and a more gradual release of the drug until 72 h. This gradual release over time in PGD nanofibers can be attributed to the distinct properties of the materials used. PCL, being hydrophobic, coupled with the hydrophilic nature of gelatin, resulted in a prolonged and sustained release of the drug, as reported in recent studies [31]. This peculiar response is also closely related to the crosslinking treatment of fibers that contributes to the more efficient retention of the drug in the fiber network, in agreement with previous studies [42]. 

From this perspective, PD and PGD nanofibers were investigated in vitro to evaluate fibroblasts’ response in the presence of DicNa. Electrospun fibers based on PCL and PCL–gelatin have been widely used for in vitro studies. Several results reported in the literature validate the use of gelatin in terms of biocompatibility, especially to promote cell adhesion [43,44]. However, no studies have focused on anti-inflammatory drugs’ impact on the in vitro and in vivo response. 

**Figure 5:**
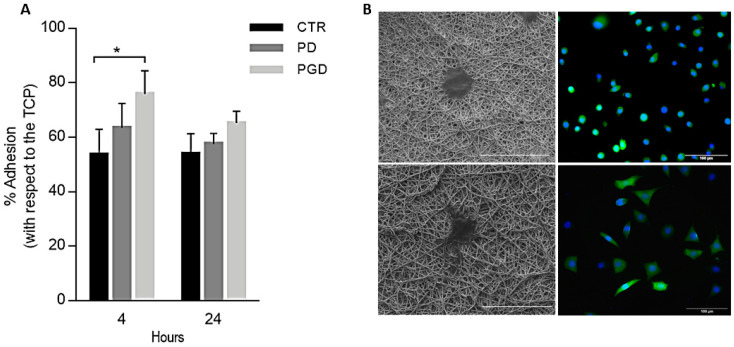
Biocompatibility assays. (**A**) Percentage of cell adhesion after 4 and 24 h. Results are presented as % of cell adhesion concerning control and cell culture plate (TCP), a statistically significant difference is represented as * *p* < 0.05. (**B**) Cell morphology captured by SEM (**left**, scale bar: 40 μm) and confocal (**right**, scale bar: 100 μm) microscopy images after 24 h.

Figure 5 shows an increase in cell adhesion after 4 and 24 h. After 4 h, the results showed a rate of more than 70% cell adhesion in the case of PGD with respect to the control, while no significant differences were observed in the case of PCL fibers. This is strictly related to the adhesive properties of gelatin, associated with the binding of the amino acid sequence RGD to integrins, as reported in previous experimental studies [23,45]. It is noteworthy that cell adhesion tends to decrease after 24 h, confirming the high sensitivity of fibroblasts to DicNa release, as reported in similar studies [46,47]. The images of cell morphology (Figure 5B) confirm a non-cytotoxic effect of DicNa on cell response, which agrees with other experimental evidence on similar systems [48]. However, it should be mentioned that different drug release profiles could affect cell interaction mechanisms, and a delay in cell growth can occur as a function of the drug released into the culture, in agreement with the inhibition mechanisms against cancer human cells reported in previous studies [49].

**Figure 6:**
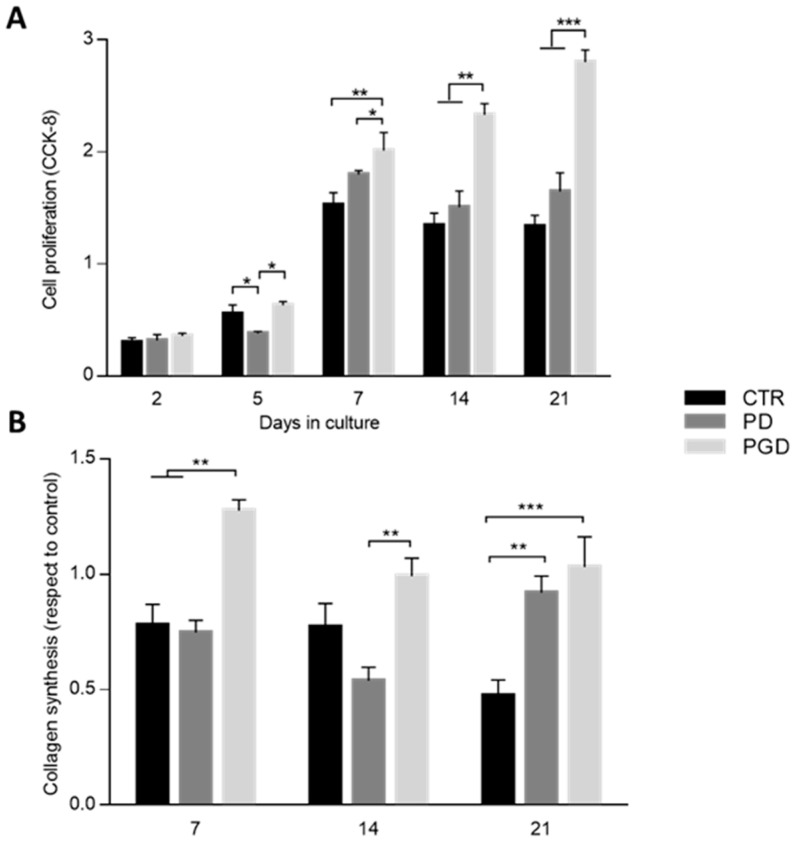
In vitro response of DicNa-loaded nanofibers: (**A**) L929 viability after 2, 5, 7, 14, and 21 days (a statistically significant difference is represented as * *p* < 0.05; ** *p* < 0.01, and *** *p* < 0.001); (**B**) Sirius red assay for fibroblast collagen synthesis after 7, 14, and 21 days (a statistically significant difference is represented as ** *p* < 0.01, and *** *p* < 0.001).

Accordingly, similar trends regarding cell viability were observed (Figure 6A). For this purpose, CCK-8 assay was performed to measure the absorbance of the reduced regent, which is proportional to living cells. After 2 days, non-significant differences were detected between the groups. An increase in the absorbance was remarked starting from day 5. This is significant in the case of PGD with respect to the CTR and PGD fibers after 7, 14 (*p* < 0.01), and 21 days (*p* < 0.001), while there was a substantial arrest in cell activity for a longer time when gelatin was not included. In light of this, further investigations were performed on the late stage of cell response for wound healing (i.e., collagen synthesis) [50]. In more detail, the collagen secreted by L929 cells was evaluated after 7, 14, and 21 days in the cell culture on CTR, PD, and PGD fibers by Sirius red assay (Figure 6B). Cells seeded onto the cell culture plate were used as a control, and all of the results were normalized. After 7 days, collagen deposition was increased—from around 0.5- to 1-fold—in the case of PGD with respect to PD and CTR, in agreement with the viability results. It is noteworthy that a greater amount of collagen is formed when vital cells tend to proliferate quickly. In the presence of gelatin, the more gradual release of DicNa does not affect the biological response of fibroblasts that are stimulated to proliferate by the bioactive signal of protein.

On the contrary, the relevant burst of DicNa release occurring in the case of PD fibers tends to temporarily inhibit the biological response of fibroblasts, with adverse effects on collagen deposition during the early cell culture times, as reported in similar studies in the literature [51,52].

Photomicrographs of cross-sections stained with H&E were used for histological evaluation after 4, 10, and 21 days of the spun materials’ implantation (CTR, PD, PGD) to analyze their contribution to regulating the wound healing process.

**Figure 7:**
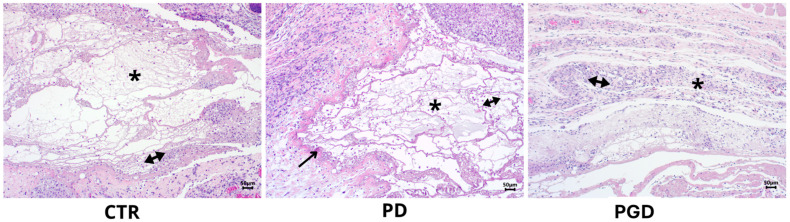
H&E staining images at 10× magnification after four days of evaluation. CTR: the double arrow corresponds to the inflammatory infiltrate. PD: the formation of a pseudocapsule is observed around the material (arrow); the double arrow indicates the formation of immune cells. PGD: the double arrow indicates the presence of cellular remains. In all cases, the asterisk is located where the material was placed.

Significant interactions between the materials and the immune system cells were observed at four days. In the control group, an amphophilic material with leukocytes inside was surrounded by a mixed inflammatory infiltrate composed mainly of foamy macrophages and epithelioid cells. This interaction is crucial as it provides insights into the immune response (Figure 7-CTR). In the case of the PD group, a pseudocapsule around the material with a fibrillar appearance was formed, with abundant macrophages in empty spaces, polymorphonuclear lymphocytes, and a few erythrocytes. The pseudocapsule presented eosinophilic thickenings with a proteinaceous appearance similar to that of immature collagen, indicating a specific immune response. Some of the cells observed in this area showed elongated clear nuclei that were intermingled with a myofibroblastic appearance, lymphocytes, plasma cells, and macrophages with an epithelioid appearance, further emphasizing the diversity of immune cells (Figure 7-PD). In contrast, in the case of the PGD group, a material with an amphophilic proteinaceous appearance was observed, including inside fragments compatible with the cellular, polymorphonuclear, and plasmatic debris occupying the empty spaces of the material, suggesting a different immune response mechanism (Figure 7-PGD) (see Figure S5 for a more detailed view of the findings at day 4).

**Figure 8:**
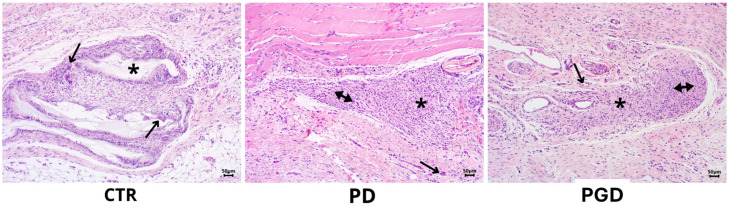
H&E staining images at 10× magnification after ten days of evaluation. CTR: the arrows correspond to foreign body giant cells. PD: immune response cells marked with the double arrow are observed, as well as the formation of blood vessels (arrow). PGD: the double arrow indicates the presence of inflammatory response cells, and the single arrow indicates the presence of blood vessels. In all cases, the asterisk is located where the material was placed.

After ten days of implantation, in the case of CTR, a space composed of fragments of amphophilic material was observed, characterized by small fragments surrounded by multinucleated giant cells of four to ten nuclei, interspersed with epithelioid cells, forming a pseudocapsule, with lymphocytes in the periphery that would correspond to the formation of a foreign body-type granuloma (Figure 8-CTR). In addition, granulomas were recognized in their periphery in the formation process, compared to the PD group. In more detail, a well-defined cluster of medium-sized cells with basophilic cytoplasm with a round nucleus and, in some cases, with tapered characteristics was observed in the space corresponding to the material. Moreover, minimal vascular neo-formation was also observed in most peripheral regions (Figure 8-PD). Contrariwise, in the case of the PGD group, a cluster of cells with an endothelioid appearance was found in the place of the fibers, in some instances discontinuous and surrounded by a few multinucleated giant cells and a few newly formed blood vessels (Figure 8-PGD). Supplementary Figure S6 provides a more detailed view of the findings at ten days.

**Figure 9:**
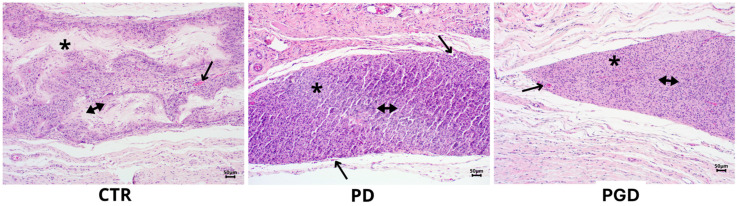
H&E staining images at 10× magnification after 21 days of evaluation. CTR: the double arrow corresponds to granulomatous inflammation, and the arrows indicate the formation of blood vessels. PD: a decrease in immune cells is observed (double arrow), as well as the formation of blood vessels (arrows). PGD: the double arrow indicates a decrease in the inflammatory response, while the single arrow indicates the formation of blood vessels. In all cases, the asterisk is located where the material was placed.

After 21 days, the formation of granulomatous inflammation was observed in the implantation area. In this process, foreign body multinucleated giant cells, epithelioid cells, and foamy macrophages interspersed with lymphocytes and plasma cells surrounded by connective tissue were involved, leading to the formation of a pseudocapsule, which is crucial in defining the immune response (Figure 9-CTR). In the case of the PD group, an island of cells with a polyhedral shape was detected, embedded in a wide eosinophilic cytoplasm with an endothelioid-type oval nucleus and small-caliber vessels inside. This unique formation provides insights into the specific immune response (Figure 9-PD). Contrariwise, in the case of the PGD group, a cluster of cells, in some instances discursive, with an endothelioid appearance was recognized in the place of the fibers, with the presence of newly formed blood vessels (Figure 9-PGD). Supplementary Figure S7 provides a more detailed view of the findings at 21 days.

## 4. Conclusions

This study investigated the in vitro and in vivo response of PCL and PCL/gelatin fibers fabricated by using the electrospinning technique and loaded with sodium diclofenac (DicNa). In vitro, the results showed increased cell adhesion and good cytocompatibility of DicNa-loaded fibers, with improved cell recognition in the case of bicomponent fibers due to the presence of gelatin. The release of DicNa did not significantly affect cellular viability and functionality (i.e., collagen synthesis). Furthermore, the in vivo study demonstrated the interaction of fibers with the immune system cells over different periods, revealing specific and diverse immune responses. The reported findings underscore the ability of PGD to modulate a lower inflammatory response than CTR and PD, providing relevant implications in the design of biomaterials for clinical applications. The proposed scaffolds could be promisingly used as drug delivery systems—alternative and complementary to current therapies—for wound healing applications.

## Figures and Tables

**Table 1:** Summary of electrospinning parameters used.

Sample	Gelatin Blending	DicNa Loading	Voltage (kV)	Flow Rate (mL/h)	Electrode Distance (mm)	Length (mm)	Spinneret Translation Rate (mm/s)
PD	-	+	15	0.1	120	200	5
PGD	+	+	13	0.5	150	-	-

## Data Availability

The data presented in this study are available in this article.

## Supplementary Materials

The following supporting information can be downloaded at: https://www.mdpi.com/article/10.3390/pharmaceutics16070925/s1, Figure S1: Surgical procedure: (A) The rat is anesthetized and sedated. Subsequently, the surgical area is disinfected, and the dorsum is divided into quadrants to mark the location of the samples. (B) An incision is made to form a flap by making a tunnel, and (C) the sample is placed as far away from the incision as possible and sutured with 5-0 nylon so as not to lose it. Finally, the tissue is faced and sutured with simple stitches; the procedure is repeated in the other samples; Figure S2: Optimization of process parameters: SEM images of DicNa-loaded PCL nanofibers for different values of voltage (13, 15, and 18 kV), flow rate (0.1, 0.5, and 1 mL/h), and electrode distance (80, 120, and 150 mm); Figure S3: Optimization of process parameters: SEM images of DicNa-loaded PCL and gelatin nanofibers for different values of voltage (13, 15, and 18 kV), flow rate (0.1, 0.5, and 1.0 mL/h), and electrode distance (80, 120, and 150 mm); Figure S4: TGA analyses: derivate curves. (A) DicNa-loaded PCL and (B) PCL/gelatin nanofibers. DicNa is referred to as the thermogram of the drug, while CTR and CTRG are reported as the controls, respectively, for unloaded PCL and PCL/gelatin nanofibers; Figure S5: H&E staining images at 20× and 40× magnification after four evaluation days. CTR: the arrow corresponds to an inflammatory response. PD: the arrow at 20× corresponds to the formation of a pseudocapsule around the material; at 40×, the arrow indicates immune cells. PGD: the arrow indicates acute inflammatory cells. In all cases, the asterisk is located where the material was placed; Figure S6: H&E staining images at 20× and 40× magnification after ten evaluation days. CTR: the double arrow indicates the inflammatory infiltrate; at 40×, the arrow points to foreign body giant cells. PD: the double arrow indicates the inflammatory infiltrate; at a higher magnification (40×), the arrow points to a foreign body giant cell. PGD: the double arrow indicates the presence of inflammatory response cells, and the single arrow indicates the presence of blood vessels. In all cases, the asterisk is located where the material was placed; Figure S7: H&E staining images at 20× and 40× magnification after 21 evaluation days. CTR: the double arrow corresponds to granulomatous inflammation; at a higher magnification, the arrow points to a foreign body giant cell. PD: a decrease in immune cells is observed (double arrow), as well as the formation of blood vessels (arrow). No foreign body giant cells were observed. PGD: the double arrow indicates a decrease in the inflammatory response; it is evident that no foreign body giant cells are present. In all cases, the asterisk is located where the material was placed.
