# Supplementary material for: Optimization of Diclofenac-Loaded Bicomponent Nanofibers: Effect of Gelatin on In Vitro and In Vivo Response
Source: Pharmaceutics. 2024 Jul 11;16(7):925. doi: 10.3390/pharmaceutics16070925 (PMC11279899; doi:10.3390/pharmaceutics16070925)
Supplement: Supplementary file 1 [file pharmaceutics-16-00925-s001.zip › pharmaceutics-3045421-supplementary.pdf]

Supplementary Data

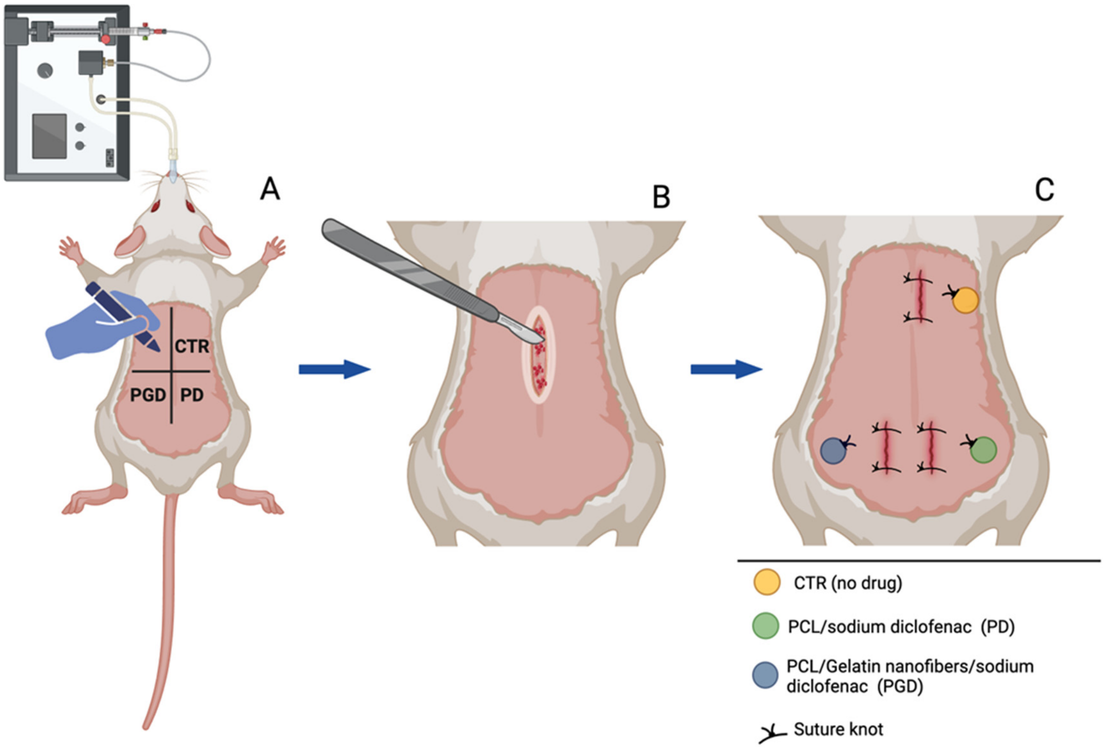

Figure S1: Surgical procedure: A) The rat is anesthetized and sedated. Subsequently, the surgical area is disinfected, and the dorsum is divided into quadrants to mark the location of the samples. B) An incision is made to form a flap by making a tunnel, and C) The sample is placed as far away from the incision as possible and sutured with 5-0 nylon so as not to lose it. Finally, the tissue is faced and sutured with simple stitches; the procedure is repeated in the other samples.

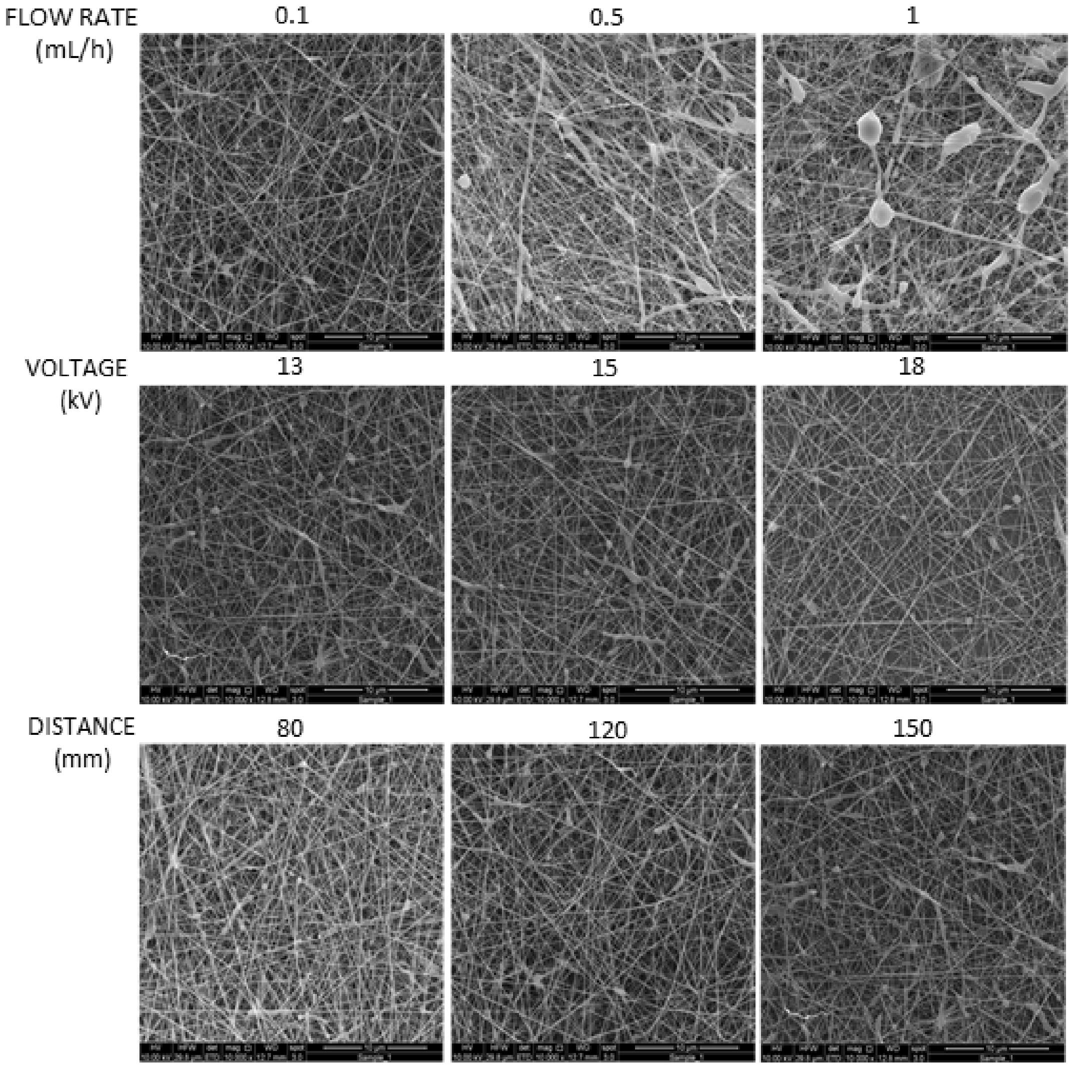

Figure S2: Optimization of process parameters; SEM images of DicNa loaded PCL nanofibers for different values of voltage (13,15 and 18 kV), flow rate (0.1, 0.5, and 1 mL/h), and electrode distance (80, 120, and 150 mm)

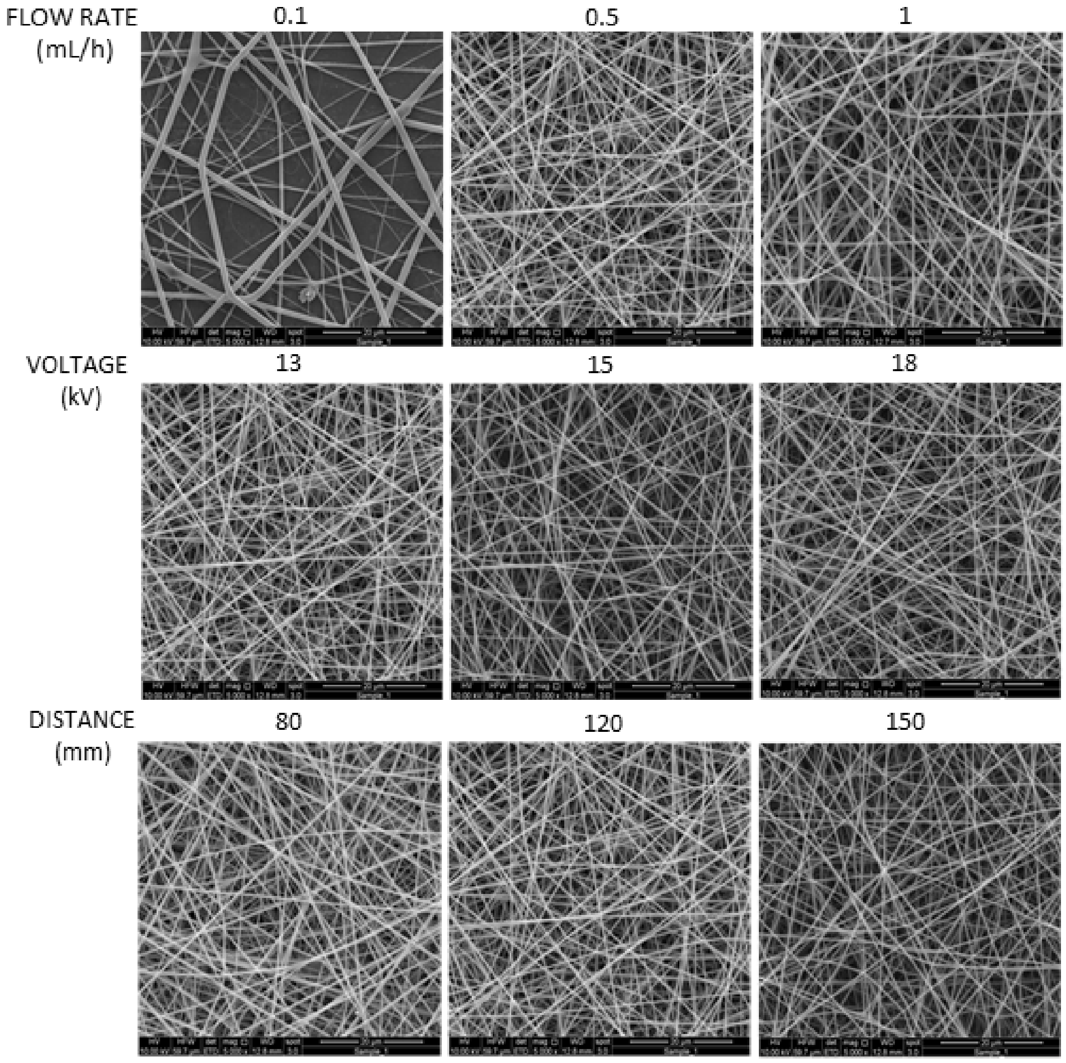

Figure S3: Optimization of process parameters; SEM images of DicNa loaded PCL and Gelatin nanofibers for different values of voltage (13,15 and 18 kV), flow rate (0.1, 0.5, and 1 mL/h), and electrode distance (80, 120, and 150 mm)

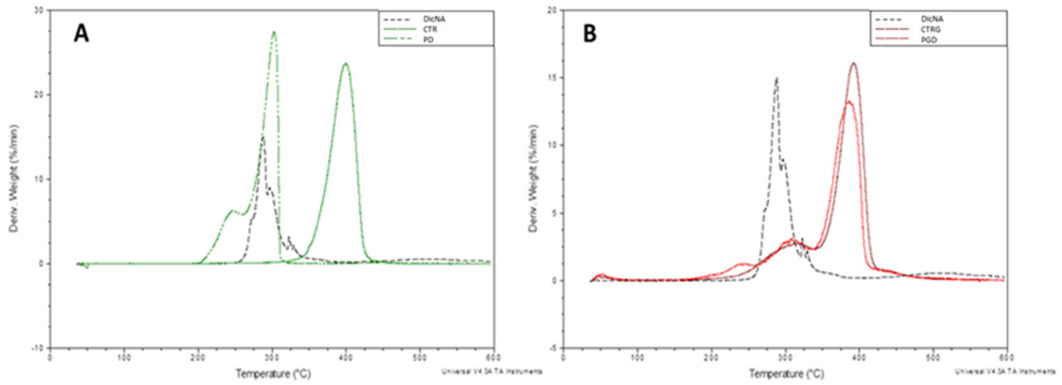

Figure S4: TGA analyses: Derivate curves - A) DicNa loaded PCL and B) PCL/Gelatin nanofibers. DicNa is referred to as the thermogram of the drug, while CTR and CTRG are reported as controls, respectively, for unloaded PCL and PCL/Gelatin nanofibers.

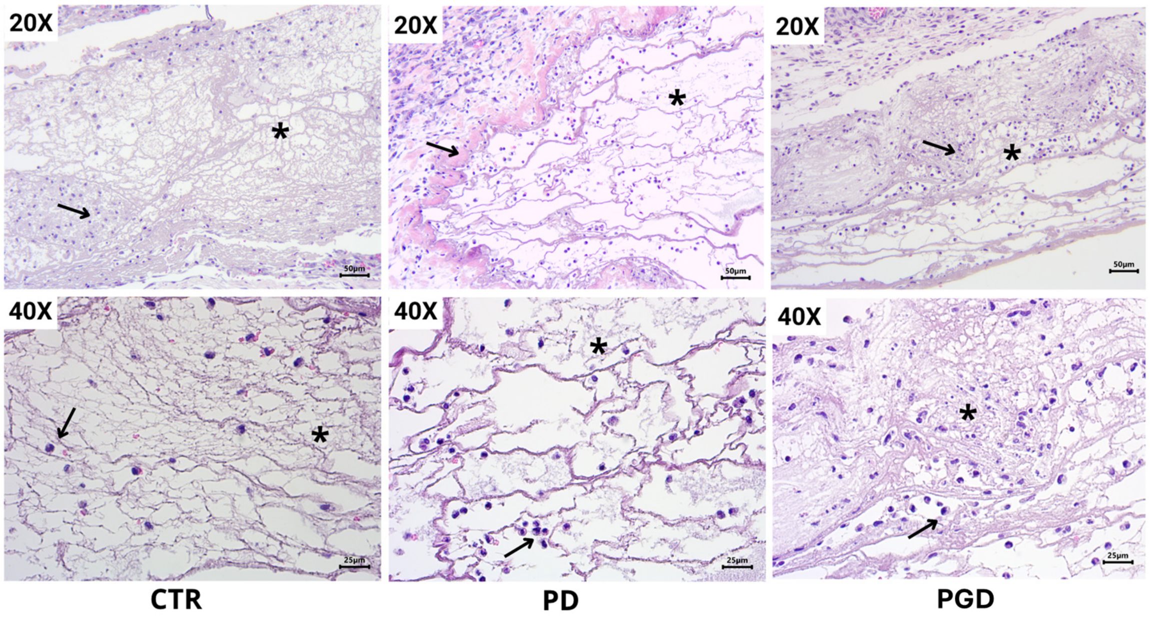

Figure S5: H&E staining images at 20x and 40x magnification after four evaluation days. CTR: The arrow corresponds to an inflammatory response. PD: The arrow at 20x corresponds to the formation of a pseudocapsule around the material; at 40x, the arrow indicates immune cells. PGD: The arrow indicates acute inflammatory cells. In all cases, the asterisk is located where the material was placed.

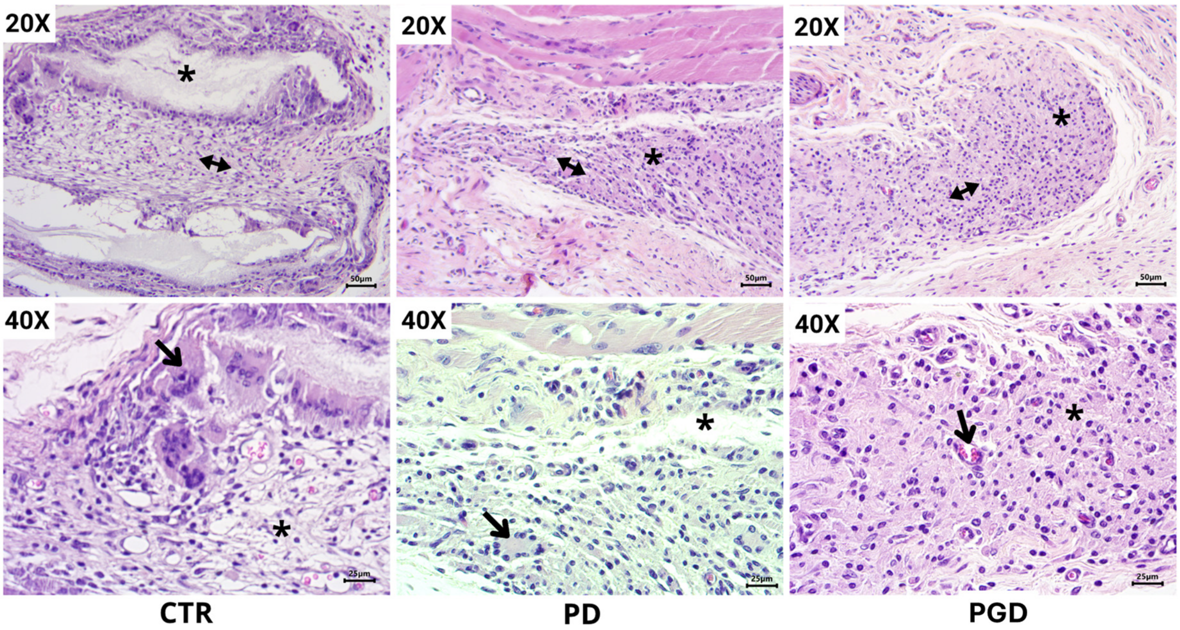

Figure S6: H&E staining images at 20x and 40x magnification after ten evaluation days. CTR: The double arrow indicates inflammatory infiltrate; at 40x, the arrow points to foreign body giant cells. PD: The double arrow indicates inflammatory infiltrate; at higher magnification (40x), the arrow points to a foreign body giant cell. PGD: The double arrow indicates the presence of inflammatory response cells, and the single arrow indicates the presence of blood vessels.
In all cases, the asterisk is located where the material was placed.

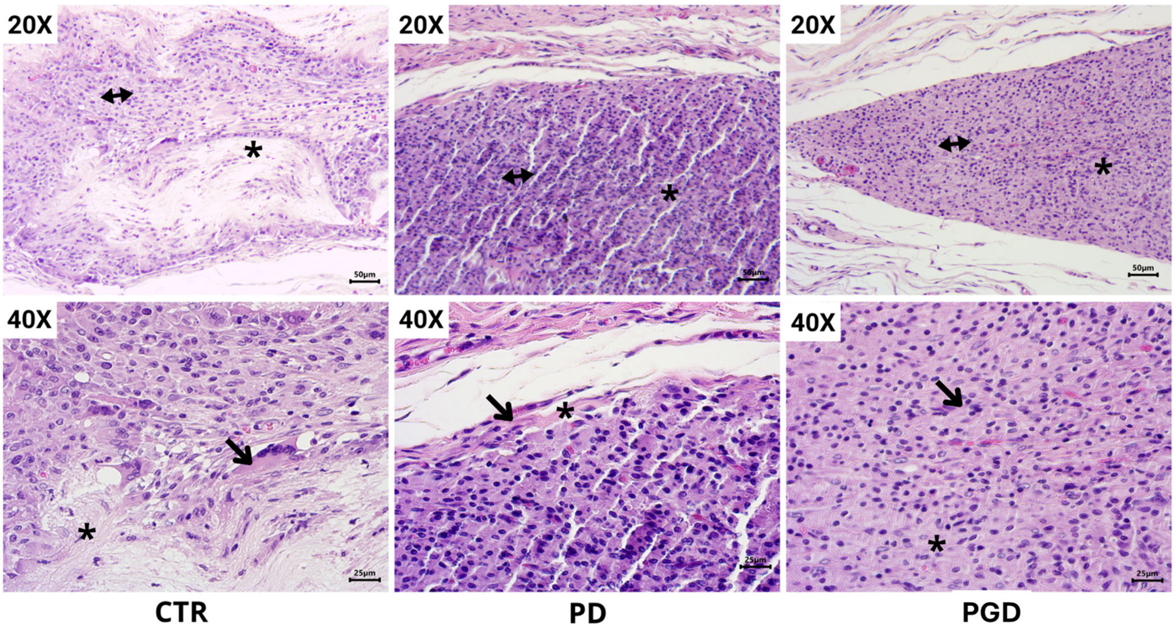

Figure S7: H&E staining images at 20x and 40x magnification after 21 evaluation days. CTR: The double arrow corresponds to granulomatous inflammation; at higher magnification, the arrow points to a foreign body giant cell. PD: A decrease in immune cells is observed (double arrow), as well as the formation of blood vessels (arrow). No foreign body giant cells were observed. PGD: The double arrow indicates a decrease in the inflammatory response; it is evident that no foreign body giant cells are present. In all cases, the asterisk is located where the material was placed.
